# Discrepancies in periodontitis classification among dental practitioners with different educational backgrounds

**DOI:** 10.1186/s12903-020-01371-5

**Published:** 2021-01-22

**Authors:** Se-Lim Oh, Ji Seung Yang, Yoon Jeong Kim

**Affiliations:** 1 Department of Advanced Oral Sciences and Therapeutics, School of Dentistry, University of Maryland, 650 West Baltimore Street, Room 4211, Baltimore, MD 21201 USA; 2 Department of Human Development and Quantitative Methodology, College of Education, University of Maryland, College Park, MD USA; 3 Department of Periodontics, School of Dentistry, Loma Linda University, Loma Linda, CA USA

**Keywords:** Periodontology, Periodontitis classification, Dental education, Dental practitioner

## Abstract

**Background:**

The 2018 classification of periodontal disease characterizes the disease with a multidimensional staging and grading system. The purpose of this multicenter study was to examine variations in periodontitis classification among dental practitioners with different postgraduate educational backgrounds at the University of Maryland School of Dentistry and the Loma Linda University School of Dentistry using the 2018 classification.

**Methods:**

This cross-sectional observational study included two cohorts: dental practitioners with periodontal backgrounds (n_1_ = 31) and those with other educational backgrounds (n_2_ = 33). The survey instrument contained three periodontitis cases presented with the guideline of the 2018 classification and a questionnaire including closed and open-ended questions. The participants were asked to review each case and to fill out the questionnaire independently. Fisher’s exact test was conducted to examine the differences in responses between the two cohorts. Polychoric correlations were calculated to examine the relation between the level of familiarity with the 2018 classification and the accuracy of the classification.

**Results:**

The distribution of item responses was significantly different between the two cohorts regarding only one item, grading for Case 1 (*p* = 0.01). No significant differences in accuracy between the two cohorts were observed except for two items, grading in Case 1 (*p* = 0.03) and staging in Case 3 (*p* = 0.04). There were no significant differences in risk factor identification for each case among the two cohorts (*p* = 1.00, Case 1; *p* = 0.22, Case 2). Staging in Case 3

($\widehat{\rho }$ = 0.52) and risk factor identification in Case 2 ($\widehat{\rho }$= 0.32) were significantly correlated with familiarity with the 2018 classification.

**Conclusion:**

A fair level of agreement in periodontitis classification was observed among dental practitioners with different educational backgrounds when the 2018 classification was used. The periodontal cohort showed better agreement levels and partially better accuracy. Risk factor identification for periodontal disease was difficult regardless of the educational background.

## Background

Periodontitis is a progressive inflammatory disease and continues to be a major etiology for tooth loss [1]. According to the data from the National Health and Nutrition Examination Survey (NHANES) 2009–12, the prevalence of periodontitis is 46%, with nearly 9% of severe cases of periodontitis in the US population aged ≥ 30 years [2]. Providing comprehensive periodontal treatment in a timely manner is important to save natural teeth.

Diagnosis is the first important decision made by a practitioner for the patient and guides treatment planning. Moreover, diagnosis reflects the practitioner’s knowledge, clinical insight, and problem-solving skills and it depends on two abilities: skill in detection during the examination and knowledge of the definition and criteria applied for identification of a disease or condition [3]. Dental practitioners have noticed that there are obvious differences in the presentation of periodontal diseases among patients and have attempted to classify periodontal diseases [4]. Classification systems have been developed to aid diagnosis and treatment decisions [5], yet even with these systems in place, considerable disagreements in diagnosis and classification have been reported among dental practitioners when using the 1999 classification of periodontal disease [6, 7]. These inconsistencies could cause over- or underestimation of the severity of periodontal disease and may lead to inappropriate treatment selection for patients.

The 1999 classification of periodontal disease has been used during the past 17 years. Nevertheless, the 1999 system has several important weaknesses, including substantial overlap and a lack of clear pathobiology-based distinction between the specified categories, diagnostic indistinctness, and difficulties in implementation [8]. While general practitioners (GPs) are patients’ initial contact for seeking periodontal treatments and a primary source for referrals to periodontists, a study found that only 62% of GPs felt confident in diagnosing aggressive periodontitis [9].

The 2018 classification was published to update the 1999 classification with scientific evidence. The diseases previously recognized as “chronic” or “aggressive” are now grouped under a single category, “periodontitis” and are further characterized based on a multidimensional staging and grading system [8, 10]. Staging is designed to categorize the severity and extent of periodontitis and is determined based on the levels of clinical attachment loss (CAL) and the percentage of radiographic bone loss (RBL) around teeth. Grading is intended to indicate the rate of disease progression. The current classification recommends that clinicians should initially assign grade B and look for specific evidence to move to grade A or C. Risk factors for periodontal disease, such as diabetes and smoking, are introduced as a grade modifier [8].

To the best of our knowledge to date, no study has investigated variations in periodontal diagnosis and classification among dental practitioners with different educational backgrounds using the current classification of periodontal disease. Ability to distinguish periodontitis Stage I and II from Stage III and IV is important for dental practitioners without periodontal educational backgrounds to recognize severity and complexity of periodontitis cases so that they can treat the mild periodontitis cases themselves and refer the severe cases to specialists.

The purpose of this multicenter study was to examine variations in periodontitis classification among dental practitioners with different postgraduate educational backgrounds at the University of Maryland School of Dentistry (UMSOD) and the Loma Linda University School of Dentistry (LLUSD) using the current classification. The findings can provide feedback on the newly developed current classification system and critical implications for dental educators in training both dental practitioners with and without a periodontal background. The following research questions (RQs) were set to make such contributions.

RQ1: Are there any significant differences in periodontitis classification between the groups of dental practitioners with or without a periodontal background when using the current classification system?

RQ2: Are there any significant differences in the accuracy of periodontitis classification between the two groups of dental practitioners in comparison with the correct stage and grade that the expert panel agreed on each case?

RQ3: Does familiarity with the current classification system influence the accuracy of the classification of each case?

## Methods

### Ethical approval

This study was conducted under a protocol approved by the institutional review board at the University of Maryland Baltimore (HP-00085364) and at Loma Linda University (5190255).

### Study design and data collection

A survey instrument in this study was developed by the principal investigator (PI), Se-Lim Oh (SO), and Yoon Jeong Kim (YK). Three periodontitis cases (Cases 1, 2, and 3) were selected by SO and YK from the patient database in the UMSOD and the LLUSD, which were considered to represent three different stages of periodontitis. The three cases were “real-life” cases without any alterations or modifications in the patients’ data. Each case presentation contained a brief medical and dental history, a full-mouth periodontal chart, intraoral clinical photographs, and intraoral complete radiographs without patient personal identifiers. An expert panel, comprised with the PI (SO) and two board-certified periodontists, reviewed the three cases in this study and drew a consensus agreement on Stage and Grade for each case.

The survey instrument in this study contained these three periodontitis cases, the guideline of the current periodontitis classification, and a questionnaire including closed and open-ended questions. The guideline for the new classification used in this study was taken from Tonetti et al. [11]. The three cases are available as the Additional file 1: Supplementary Material. Table 1 shows the questionnaire used in this study and the expected answers for each case.

**Table 1:** Questionnaire used in this study and expected answers for each case

**I am a postgraduate resident** (or a faculty) in _______ 1. Periodontics 2. Advanced General Dentistry 3. Endodontics 4. Prosthodontics 5. Orthodontics
**How familiar** are you with the 2018 periodontal disease classification? 1. I am not aware that the 2018 periodontal classification is available 2. I am aware of the 2018 periodontal classification, but I am not using it in my practice 3. I am aware of the new periodontal classification and am in the process of integrating it into my practice 4. I am diagnosing patients exclusively using the 2018 periodontal classification
**Case 1**
1) Based on the clinical and radiographic evaluation, diagnose this patient’s periodontal disease with Stage and Grade *Expected Answer: Stage III, Grade C*
2) Does this patient have any risk factors for periodontal disease? If yes, please indicate the risk factor(s) *Expected Answer: None*
**Case 2**
1) Based on the clinical and radiographic evaluation, diagnose this patient’s periodontal disease with Stage and Grade *Expected Answer: Stage IV, Grade B*
2) Does this patient have any risk factors for periodontal disease? If yes, please indicate the risk factor(s)
*Expected Answer: Diabetes and smoking*
**Case 3**
1) Based on the clinical and radiographic evaluation, diagnose this patient’s periodontal disease with Stage and Grade *Expected Answer: Stage II, Grade B*

This cross-sectional observational study included two cohorts: dental practitioners with periodontal backgrounds and dental practitioners with other educational backgrounds. Faculty and postgraduate (PG) dental residents in periodontics at the UMSOD and the LLUSD and all PG dental residents in other dental disciplines at the UMSOD participated in this survey. PG dental residents had graduated from dental schools and entered additional specialty training programs. The participants were asked to review each case with the provided guideline and fill out the questionnaire independently with no time restrictions. No personal identifiable information of the participant was associated with responses, and only codes for subgroups were used. Data collection was conducted from July 2019 to January 2020.

### Study sample

Table 2 summarizes the participants from each dental school. A total of 64 participants were included. In the periodontal cohort (n_1_ = 31), faculty and PG residents from the two schools were recruited, while PG residents only from the UMSOD were recruited in the nonperiodontal cohort (n_2_ = 33).

**Table 2:** Summary of the participants (the number of participants)

	Periodontal background	Nonperiodontal background
School (n)	UMSOD (12) LLUSD (19)	UMSOD (33)
Composition (n)	Faculty (13) PG residents (18)	All PG residents: Advanced general dentistry (7) Prosthodontics (9) Endodontics (6) Orthodontics (11)

UMSOD = University of Maryland School of Dentistry; LLUSD = Loma Linda University School of Dentistry; PG = postgraduate

The priori power analysis revealed that the current sample size allows us to detect the medium to large effect size of 0.3 to 0.4 with an adequate power of 0.8 to 0.85 when either chi-square or Fisher’s exact test is used.

### Statistical analysis

After examining descriptive statistics, Fleiss’ kappa and Kendall’s coefficient of concordance (W) were first calculated to gauge the level of agreement and concordance over eight items among the overall participants as well as within each cohort. To examine the differences in the responses between the two cohorts of interest with respect to each item, Fisher’s exact test was conducted [12, 13].

To examine the differences in the accuracy of the responses between the two cohorts, the responses were dichotomously scored. The “correct” diagnosis and classification were determined by the expert panel members whose agreement reached 100%. Then, the scored responses were compared between the two different cohorts using Fisher’s exact tests with respect to each item.

To examine the relation between the level of familiarity with the current classification and the accuracy of the classification, polychoric correlations were calculated to address the nature of categorical responses that are ordinal. All analyses were conducted using R with the packages, polychor, psych, and irr [14–16]. A *p *value ≤ 0.05 was considered significant.

## Results

No responders answered the extent with the stage for each case. Therefore, the extent was not included in the analysis.

Table 3 exhibits the number of participants and the distribution for levels of the acquaintance with the utilization of the current classification in each cohort. Seventy-four percent of the periodontal cohort exclusively used the current classification, while nearly 79% of the nonperiodontal cohort answered as either not aware of or not using the current classification. As such, the distributions of responses appear significantly different between the two groups (Fisher's exact test; *p* < 0.001).

**Table 3:** Distribution for levels of the acquaintance and the utilization of the 2018 classification among the participants (Fisher's exact test *p* < *0.001*)

Participants	1 (not aware)	2 (aware but not using)	3 (in the process of integrating into practice)	4 (exclusively using the 2018 classification)
Practitioners with a periodontal background (n_1_ = 31)	0 (0%)	1 (3%)	7 (23%)	23 (74%)
Practitioners with other educational backgrounds (n_2_ = 33)	9 (27.3%)	17 (51.5%)	7 (21.2%)	0 (0%)
Overall (N = 64)	9 (14.1%)	18 (28.1%)	14 (21.9%)	23 (35.9%)

% is the row percentage, rounded up to the first digit after the decimal point

**Fig. 1:**
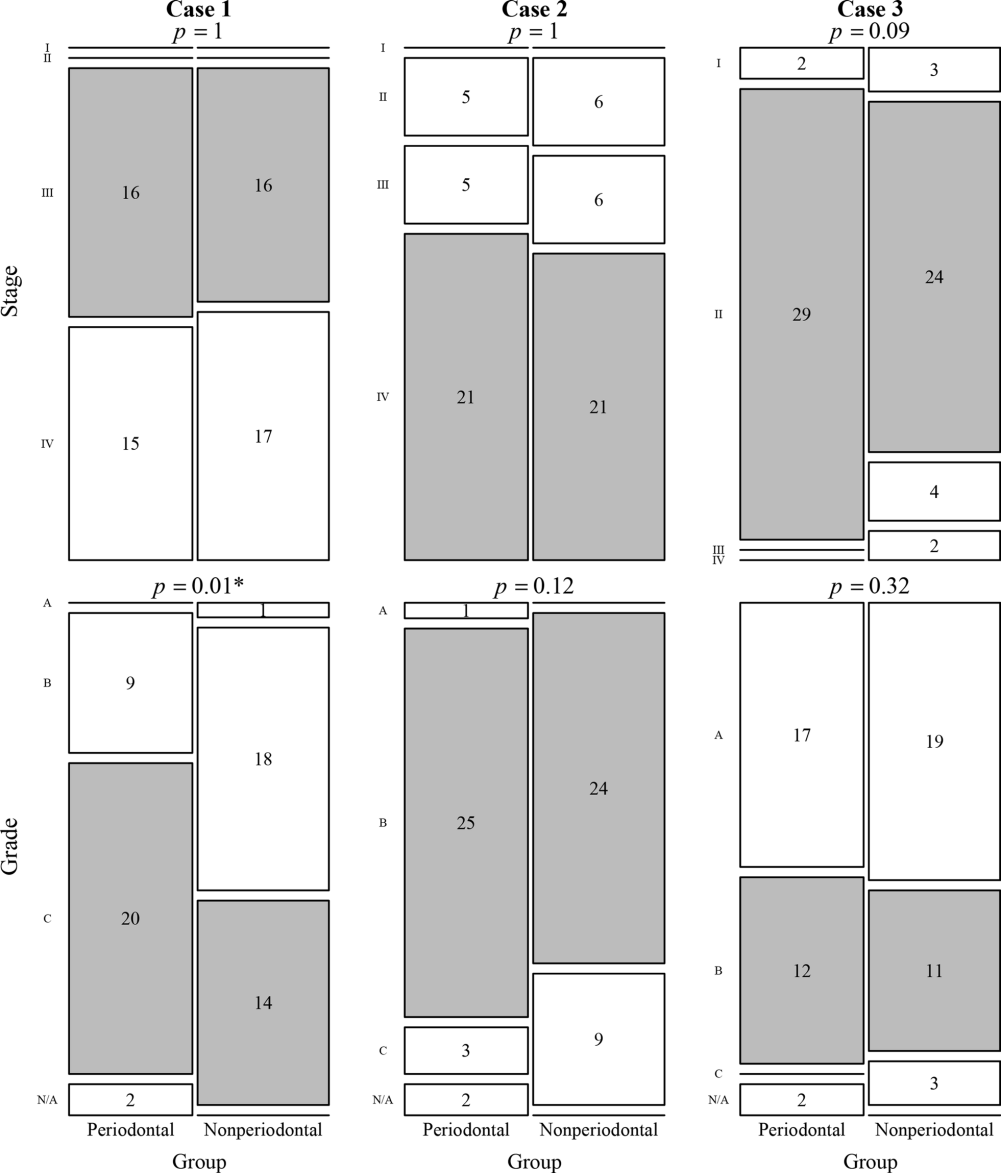
Mosaic plot of the staging and grading for each case among the participants (total sample = 64; participants with a periodontal background = 31; participates with a nonperiodontal background = 33). Note: The p-value for Fisher’s exact test is reported on top of each panel. The height of each box represents the proportion of each response category within a group, and the numbers in boxes represent the corresponding counts. The correct response categories are shown in gray. N/A refers to “not answered”

RQ1. Figure 1 shows the distributions of item responses on staging and grading with respect to each case among the participants. Fisher’s exact test for each item revealed that the distribution of item responses was significantly different between the periodontal and nonperiodontal cohorts with respect to only one item, the responses on grading for Case 1 (Fisher’s exact test; *p* = 0.01).

Across a total of six staging and grading items in three cases, the overall sample of 64 participants exhibited fair (0.21 < κ < 0.40) to moderate (0.41 < κ < 0.60) levels of agreement ($\widehat{\kappa }$ = 0.34, z = 61.1, *p* < 0.001) and concordance (W = 0.61, df = 5, ${\chi }^{2}$=195, *p* < 0.001). Among the periodontal cohort, the agreement and concordance levels were higher ($\widehat{\kappa }$ = 0.41 z = 37.2, *p* < 0.001; W = 0.68, df = 5, ${\chi }^{2}$=105, *p* < 0.001) than the corresponding coefficients calculated from the nonperiodontal cohort ($\widehat{\kappa }$=0.28, z = 25.6, *p* < 0.001; W = 0.57, df = 5, ${\chi }^{2}$=93.6, *p* < 0.001). In particular, the difference in $\widehat{\kappa }$ estimates between the two cohorts was statistically significant at the *p* = 0.05 level, indicating that a significantly higher level of agreement was observed within the periodontal cohort.

RQ2. The correct classification rates for each case are reported in Table 4 along with the correct identification of risk factors for periodontal disease. The scored data analysis revealed that fewer than 50% of the total participants were successful on three out of eight items: risk factor identification for Case 1, risk factor identification for Case 2, and grade for Case 3. Generally, there were no significant differences in accuracy between the two cohorts except for two items; grading in Case 1 and staging in Case 3. For these items, the performance of the periodontal cohort was significantly better than that of the nonperiodontal cohort (Fisher’s exact test; *p* = 0.03 for grading in Case 1 and *p* = 0.04 for staging in Case 3). There were no significant differences in the recognition of risk factors for each case among the two cohorts (Fisher’s exact test; *p* = *1.00* for Case 1, *p* = *0.22* for Case 2).

**Table 4:** Accuracy in the periodontitis classification and recognition of risk factors for periodontal disease among the participants (%)

	Periodontal (n_1_ = 31)	Nonperiodontal (n_2_ = 33)	Overall (N = 64)	*p*-value*
Case 1, Stage	52	48	50	1.00
Case 1, Grade	72	42	56	**0.03**
Case 1, Risk factor	32	30	**31**	1.00
Case 2, Stage	68	64	66	0.80
Case 2, Grade	81	73	77	0.56
Case 2, Risk factor	48	30	**39**	0.22
Case 3, Stage	94	73	83	**0.04**
Case 3, Grade	39	33	**36**	0.80

^*^ Fisher's exact test

RQ3. The polychoric correlations between familiarity with the current classification and each scored item are presented in Table 5. Two statistically significant correlations were found between familiarity with the current classification and staging in Case 3 ($\widehat{\rho }$ = 0.52, SE = 0.16, z = 3.25) and risk factor identification in Case 2 ($\widehat{\rho }$ = 0.32, SE = 0.18 z = 2.25). Given that the level of familiarity with the current classification was confounded by the periodontal background, we also calculated the same correlations within each group. Once the periodontal background was controlled for, some of the correlations were negative for the nonperiodontal cohort, in which none of the participants were using the current classification in their practice. This indicates who were not aware of or not using the guideline of the current classification performed better in periodontitis classification when the guideline was provided.

**Table 5:** Correlation between familiarity with the 2018 classification and participant performance on each item (Polychoric correlation coefficient)

	Periodontal cohort (n_1_ = 31)	Nonperiodontal cohort (n_2_ = 33)	Overall (N = 64)
Case 1, Stage	0.38	**−** **0.60**	**−** 0.08
Case 1, Grade	0.17	**−** **0.49**	0.16
Case 1, Risk factor	0.86	**−** 0.33	0.06
Case 2, Stage	0.44	**−** **0.55**	**−** 0.06
Case 2, Grade	0.02	0.25	0.21
Case 2, Risk factor	0.41	0.44	**0.32**
Case 3, Stage	0.15	0.34	**0.52**
Case 3, Grade	0.11	0.06	0.12

## Discussion

Although researchers have made efforts to develop new technologies to improve diagnostic ability [17–20], periodontal diagnosis and classification are still formulated based on clinical and radiographic data collected by individual practitioners. A practitioners’ ability to interpret and integrate the data obtained and critical thinking skills for clinical reasoning yields meaningful periodontal decisions [21]. The purpose of this study was to examine the variations in periodontitis classification among dental practitioners with different postgraduate educational backgrounds using the current classification.

We found that there was a fair level of agreement among all participants and the agreement level was higher among the periodontal cohort than the nonperiodontal cohort when the current classification was employed (Fig. 1). Although fair to moderate agreement was obtained, the accuracy was not at the satisfactory level, ranging from 31 to 83% at the most (Table 4). Even for the most straightforward case from the investigators’ point of view (Case 3), the grading accuracy was 36%. For only two items out of eight, the periodontal cohort demonstrated significantly better accuracy in periodontitis classification (grading for Case 1 and staging for Case 3).

Grading, especially for new patients, could be challenging because dental practitioners often do not have previous periodontal records, such as CAL or RBL. Calculating the amount of CAL or RBL over 5 years, which was suggested as direct evidence in the current classification [8], is difficult. Instead, dental practitioners often use the %bone loss/age index as indirect evidence. The goal of incorporating grading is to estimate the future risk of periodontitis progression and responsiveness to standard therapeutic principles to guide the intensity of therapy and monitoring [11]. Grading is also designed for estimating the potential impact of systemic health on periodontitis to promote comanagement of patient health with medical teams [11]. Having an accurate grade influences the management of the case, including the treatment goal, strategy, treatment modalities and/or sequence.

Identification of risk factors for periodontal disease is also difficult regardless of the educational background, as indicated by the low level of accuracy and the lack of a significant difference in the recognition of risk factors for the two cases (Cases 1 and 2) among the two cohorts. Risk factors, when present in an individual, increase the chance of developing the disease by modifying host responses to the etiology, bacterial plaque, in periodontal disease [22]. Although the guideline stated diabetes and smoking as a risk factor, many responders answered hypertension as a risk factor for periodontitis. This may indicate that the concept of risk factors for periodontal disease is not well understood among many participating dental practitioners in this study. Since risk factors play a role as grade modifiers in the grading system, emphasis on risk factors for periodontal disease in dental education is recommended.

Since the current classification was published in 2018, many dental practitioners with other education backgrounds were not familiar with the classification. Interestingly, when the periodontal background was controlled for, some of the correlations between familiarity and accuracy for staging and grading were even negative for the nonperiodontal cohort (Table 5). This implies that dental practitioners who were not aware of or were not using the current classification performed well in periodontitis classification when the guidelines were provided. It is noteworthy that this result is only generalizable for the population of dental practitioners who have nonperiodontal backgrounds and have never used the current classification. Among this population, the familiarity level is not a key to classify periodontitis cases.

A recent publication emphasized that Stage is a patient-based, not a tooth-based concept [23]. The authors acknowledged that there is a gray zone for the clinicians to use clinical judgement for certain patient cases. Therefore, obtaining all necessary information including patient’s medical history, radiographs, and a full mouth periodontal charting is important [23]. The critical information for clinicians to determine staging and grading for patients is CAL, etiologies for CAL, % RBL, and patient’ age, which dental practitioners should be able to interpret.

The generalizability of our study results is limited due to a few factors. The sample size was relatively small. Only PG dental residents from the UMSOD were included in the nonperiodontal cohort while the periodontal cohort was comprised from both universities. All dental education for undergraduate and postgraduate training in the US should follow the Commission on Dental Accreditation guideline. Therefore, the training for specialists in both universities is similar although they are not the exact same; PG periodontics programs in both universities use the current periodontitis classification. The PG dental residents from the UMSOD in the nonperiodontal cohort was included because the PI confirmed that they did not receive the formal education on the current periodontitis classification, while PG dental residents in some programs from the LLUSD have been implementing the current classification in their training. The number of cases and items for each case were small in the questionnaire. While the three patient cases were meant to represent different scenarios with the investigators’ intention, more cases and items related to each case are necessary to cover contents related to periodontal diagnosis and classification such as clinical and radiographic data assessments, local contributing factors for periodontal disease, and occlusal evaluation.

## Conclusion

A fair level of agreement in periodontitis classification was observed among dental practitioners with different educational backgrounds when the 2018 classification was used. The periodontal group showed better agreement levels and partially better accuracy. Identification of risk factors for periodontal disease was difficult regardless of the educational background.

## Supplementary Information

**Additional file 1.** Three periodontitis cases included in the survey instrument.

## Data Availability

The datasets used and analyzed in this study are available from the corresponding author upon reasonable request.

## Abbreviations

CAL: Clinical attachment loss
RBL: Radiographic bone loss
FGM: Free gingival margin
CEJ: Cementoenamel junction
PD: Probing depth
Bleed/S: Bleeding/suppuration
BP: Blood pressure
ASA: American Society of Anesthesiologists Physical Status Classification

## References

[1] Rozier RG, White BA, Slade GD (2017). Trends in oral diseases in the U. S. population. J Dent Educ..

[2] Eke PI, Dye BA, Wei L (2015). Update on prevalence of periodontitis in adults in the United States: NHANES 2009 to 2012. J Periodontol.

[3] Jutel A (2009). Sociology of diagnosis: a preliminary review. Sociol Heal Illn.

[4] Highfield J (2009). Diagnosis and classification of periodontal disease. Aust Dent J.

[5] Armitage GC (2000). Periodontal diagnoses and classification of periodontal diseases. Periodontology.

[6] Lanning SK, Pelok SD, Williams BC (2005). Variation in periodontal diagnosis and treatment planning among clinical instructors. J Dent Educ.

[7] Lane BA, Luepke P, Chaves E (2015). Assessment of the calibration of periodontal diagnosis and treatment planning among dental students at three dental schools. J Dent Educ.

[8] Papapanou PN, Sanz M, Buduneli N (2018). Periodontitis: Consensus report of workgroup 2 of the 2017 world workshop on the classification of periodontal and peri-implant diseases and conditions. J Periodontol.

[9] Darby IB, Angkasa F, Duong C (2005). Factors influencing the diagnosis and treatment of periodontal disease by dental practitioners in Victoria. Aust Dent J.

[10] Caton J, Armitage G, Berglundh T (2018). A new classification scheme for periodontal and peri-implant diseases and conditions – Introduction and key changes from the 1999 classification. J Clin Periodontol.

[11] Tonetti MS, Greenwell H, Kornman KS (2018). Staging and grading of periodontitis: framework and proposal of a new classification and case definition. J Periodontol.

[12] Fisher RA (1922). On the interpretation of χ^2^ from contingency tables, and the calculation of P. J R Stat Soc.

[13] Cochran WG (1952). The χ^2^χ^2^ test of goodness of fit. Ann Math Stat.

[14] Gamer M, Lemon J, Fellows I, Singh P. Various coefficients of interrater reliability and agreement. Published 2019. https://cran.r-project.org/package=irr

[15] Fox J. Polychoric and polyserial correlations. Published 2019. https://cran.r-project.org/package=polycor

[16] Revelle W. Procedures for psychological, psychometric, and personality research. Published 2020. https://cran.r-project.org/package=psych

[17] Loesche WJ, Bretz WA, Lopatin D (1990). Multi-center clinical evaluation of a chairside method for detecting certain periodontopathic bacteria in periodontal disease. J Periodontol.

[18] Yano K, Takamatsu N, He T, Umeda M, Ishikawa I (1996). Evaluation of non radioactive DNA probe (Affirm DP) for detecting periodontopathic bacteria. Kokubyo Gakkai Zasshi.

[19] Persson GR, De Rouen TA, Page RC (1990). Relationship between gingival crevicular fluid levels of aspartate aminotransferase and active tissue destruction in treated chronic periodontitis patients. J Periodontal Res.

[20] Chaudhari AU, Byakod GN, Waghmare PF, Karhadkar VM (2011). Correlation of levels of interleukin-1β in gingival crevicular fluid to the clinical parameters of chronic periodontitis. J Contemp Dent Pract.

[21] John V, Lee S-J, Prakasam S, Eckert GJ, Maupome G (2013). Consensus training: an effective tool to minimize variations in periodontal diagnosis and treatment planning among dental faculty and students. J Dent Educ.

[22] Papapanou P (1998). Risk assessments in the diagnosis and treatment of periodontal diseases. J Dent Educ.

[23] Kornman KS, Papapanou PN (2020). Clinical application of the new classification of periodontal diseases: Ground rules, clarifications and “gray zones”. J Periodontol.

