# Supplementary material for: Discrepancies in periodontitis classification among dental practitioners with different educational backgrounds
Source: BMC Oral Health. 2021 Jan 22;21:39. doi: 10.1186/s12903-020-01371-5 (PMC7821642; doi:10.1186/s12903-020-01371-5)
Supplement: Supplementary file 1 — Additional file 1. Three periodontitis cases included in the survey instrument. [file 12903_2020_1371_MOESM1_ESM.pdf]

Case A

- A patient (age 40-49 years) presented with a missing tooth.
- The chief complaint was to fill the space.
- Upon medical history taking, the patient had hypertension.
- Her last dental office visit was 1 year ago to extract one of the maxillary central incisors.
- The patient brushes 3 times a day and does not have any pain this time.

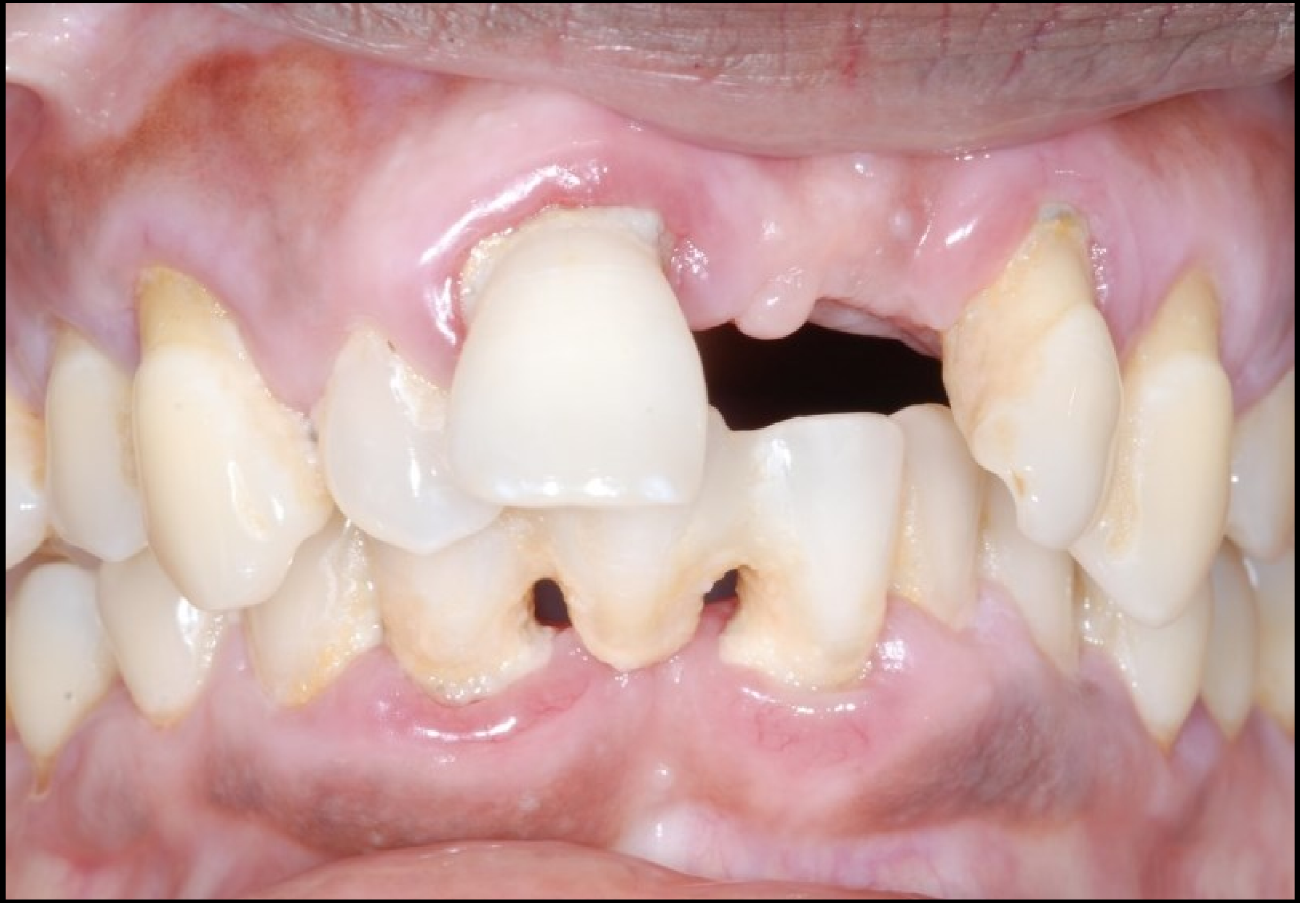

Furcation	2	2												3	2
FGM-CEJ (Facial)	2 2 2	0 0 0	2 3 2	1 2 1	1 2 1	3 3 3	-2 -1 -2	1 1 1		3 3 3	2 3 2	0 1 0	0 1 0	2 2 2	1 1 1
PD	5 5 5	4 4 5	7 5 6	4 3 7	7 4 7	5 2 8	5 3 4	3 4 5		4 2 4	6 3 6	9 8 8	7 3 5	5 3 8	9 8 9
CAL	7 7 7	4 4 5	9 8 8	5 5 8	8 6 8	8 5 11	3 2 2	4 5 6		7 5 7	8 6 8	9 9 8	7 4 5	7 5 10	10 9 10
Bleed/S	B B B	B B B	B B B	B B B	B B B	B B	B B	B B B		B B B	B B	B B B	B B B	B B B	B B B
**Tooth #**	**1**	**2**	**3**	**4**	**5**	**6**	**7**	**8**	**9**	**10**	**11**	**12**	**13**	**14**	**15**
Bleed/S	B B B	B B B	B B B	B B B	B B B	B B B	B B	B B B		B B B	B B B	B B B	B B B	B B B	B B B
CAL	6 5 5	5 8 6	9 9 9	8 7 9	8 8 7	8 8 8	4 2 4	8 5 9		6 7 9	9 8 9	9 8 7	8 5 3	6 5 7	6 5 6
PD	6 5 5	5 8 5	7 6 7	7 5 8	8 8 7	6 6 6	4 2 4	5 2 6		4 5 7	7 6 7	9 8 6	8 5 3	5 4 6	6 5 6
FGM-CEJ (palatal)	0 0 0	0 0 1	2 3 2	1 2 1	0 0 0	2 2 2	0 0 0	3 3 3		2 2 2	2 2 2	0 0 1	0 0 0	1 1 1	0 0 0
Mobility	1	1	2	1	1	1	1	2		2	2	3	1	2	2
Furcation		2 2	3 3											1 3	2 2

Furcation	2												2		
FGM-CEJ (lingual)	1 1 1		1 1 0	0 0 0	0 0 0	1 1 1		1 1 1	1 1 1	0 0 0	0 0 0	0 0 0	1 1 1	1 1 1	0 0 0
PD	8 4 5		3 3 4	5 3 4	3 1 4	4 1 3		2 2 2	2 2 2	4 2 3	5 4 4	4 3 5	8 6 7	8 8 8	7 4 4
CAL	9 5 6		4 4 4	5 3 4	3 1 4	5 2 4		3 3 3	3 3 3	4 2 3	5 4 4	4 3 5	9 7 8	9 9 9	7 4 4
Bleed/S	B B B		B B B	B B	B	B B		B B B	B B B	B B	B B	B B	B B B	B B B	B B B
**Tooth #**	**31**	**30**	**29**	**28**	**27**	**26**	**25**	**24**	**23**	**22**	**21**	**20**	**19**	**18**	**17**
Bleed/S	B B B		B B B		B B	B B B		B B B	B B B	B B	B B	B B	B B B	B B B	B B B
CAL	7 6 7		5 4 4	2 2 2	4 3 4	5 5 5		4 5 6	4 2 5	5 2 5	4 2 4	4 3 5	8 4 9	5 7 5	8 6 5
PD	5 4 5		4 3 4	2 2 2	4 3 4	4 4 4		3 4 5	4 2 5	5 1 5	4 1 4	4 2 5	7 3 8	4 6 4	7 5 4
FGM-CEJ (facial)	2 2 2		1 1 0	0 0 0	0 0 0	1 1 1		1 1 1	0 0 0	0 1 0	0 1 0	0 1 0	1 1 1	1 1 1	1 1 1
mobility	2		1	1	1	1		1	1	1	1	1	2	2	
Furcation	2												2	I	

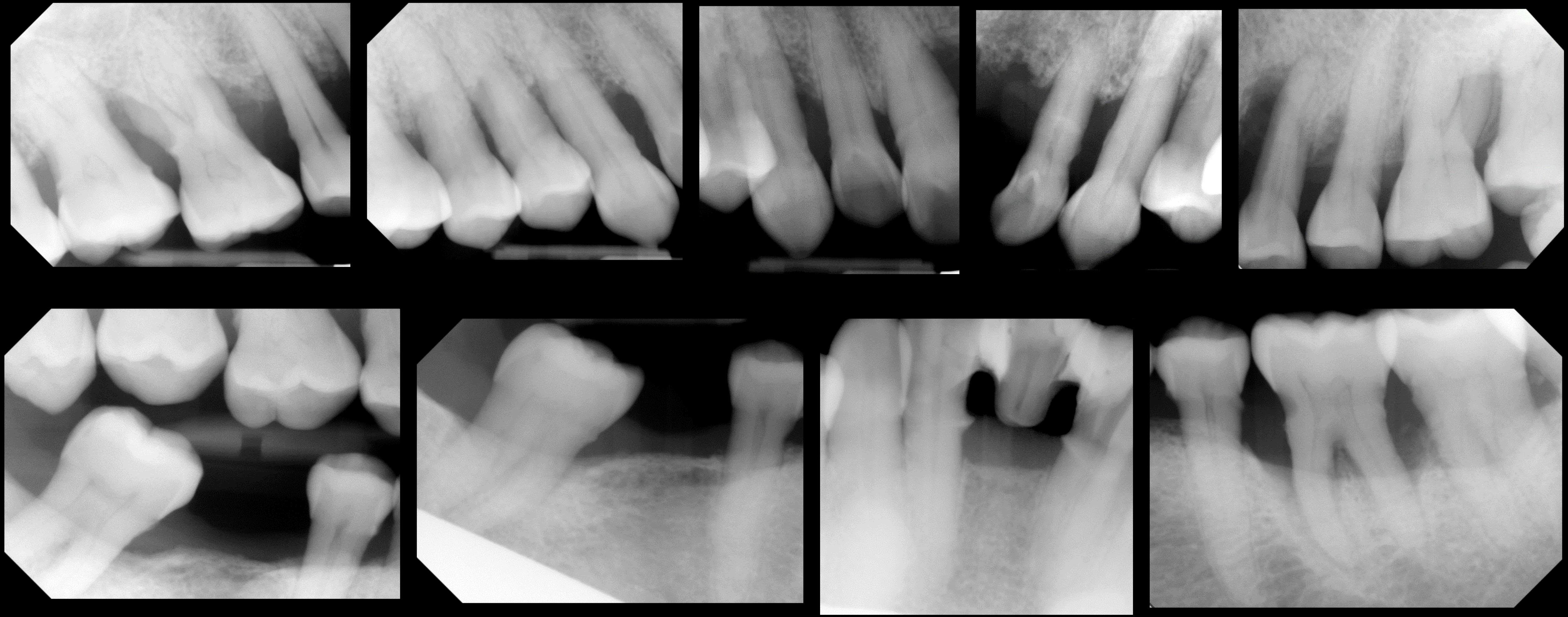

Case B

- A patient (age 60-69 years) visited the urgent care clinic and removed the maxillary right central incisor, first premolar, first and second molars.

- The chief complaint: “I want to get my teeth back on the right track"

- Medical and social history
  - Hypertension: 150/90 mm Hg
  - Type II diabetes: HbA1c <7%
  - One cigarette a week

- Medication
  - Amlodipine 10 mg
  - Atorvastatin 40 mg
  - Fluticasone 50 mcg
  - Lisinopril 40 mg
  - Levocetirizine 5 mg
  - Metformin 500 mg

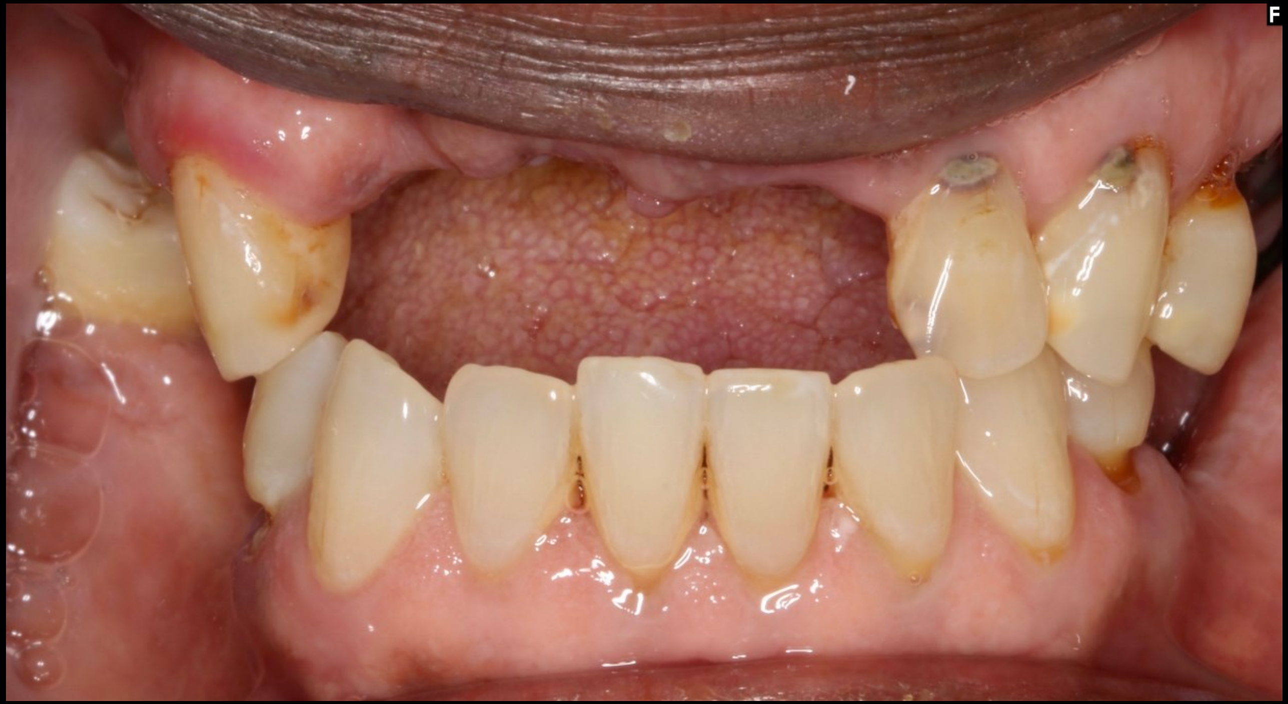

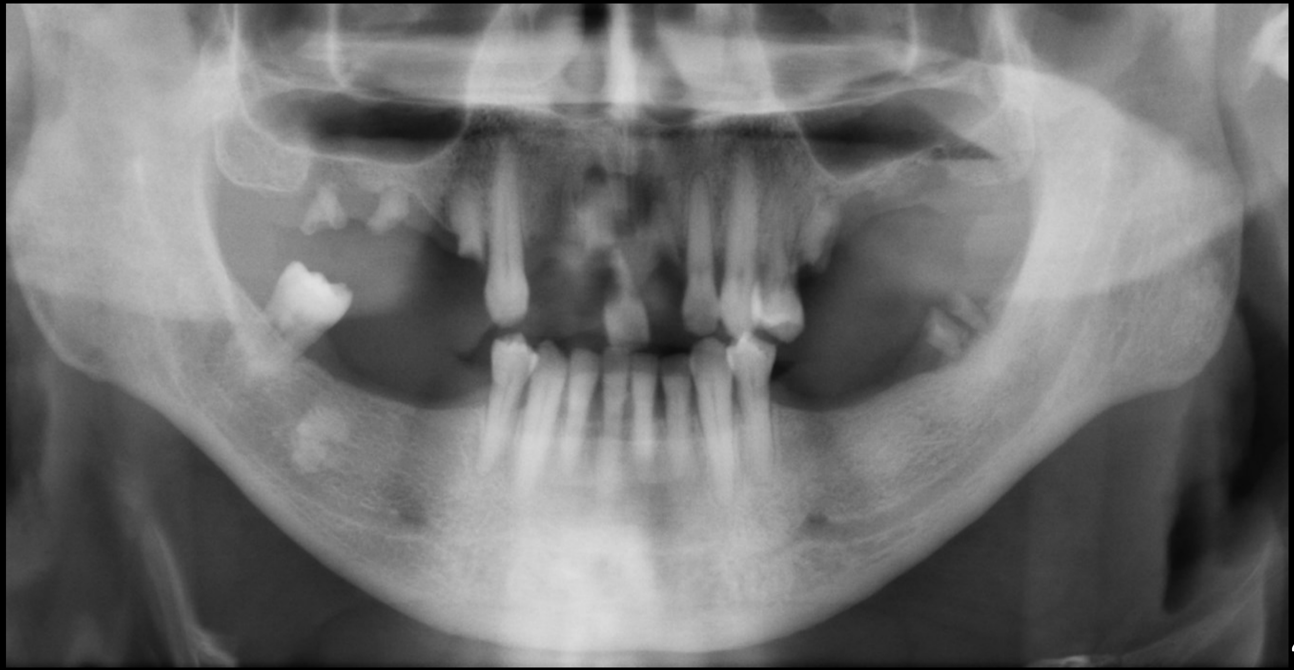

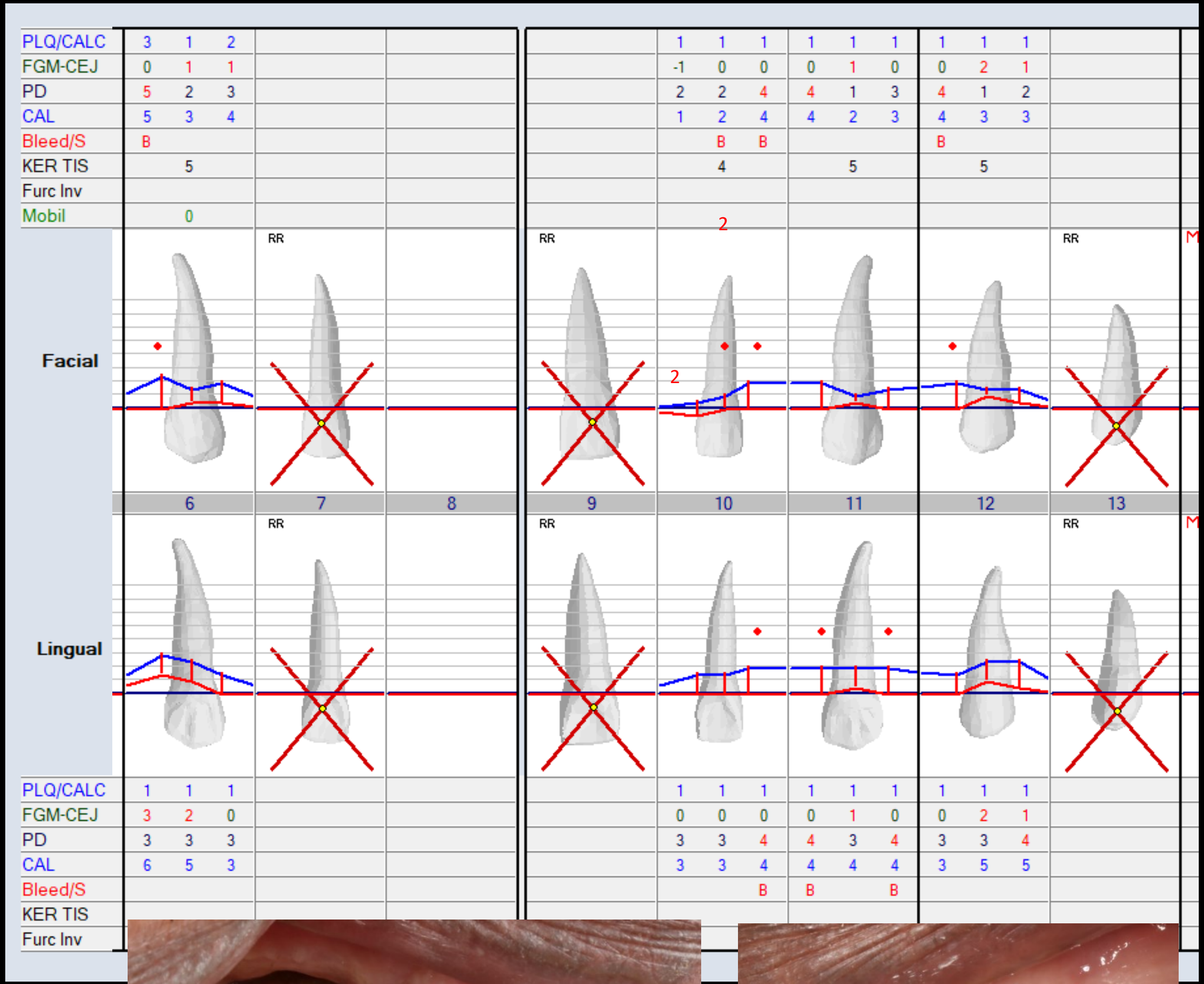

	6	7	8	9	10	11	12	13
PLQ/CALC	3 1 2				1 1 1	1 1 1	1 1 1	
FGM-CEJ	0 1 1				-1 0 0	0 1 0	0 2 1	
PD	5 2 3				2 2 4	4 1 3	4 1 2	
CAL	5 3 4				1 2 4	4 2 3	4 3 3	
Bleed/S	B				B B		B	
KER TIS	5				4	5	5	
Furc Inv								
Mobil	0							

	6	7	8	9	10	11	12	13
PLQ/CALC	1 1 1				1 1 1	1 1 1	1 1 1	
FGM-CEJ	3 2 0				0 0 0	0 1 0	0 2 1	
PD	3 3 3				3 3 4	4 3 4	3 3 4	
CAL	6 5 3				3 3 4	4 4 4	3 5 5	
Bleed/S					B	B B		
KER TIS								
Furc Inv								

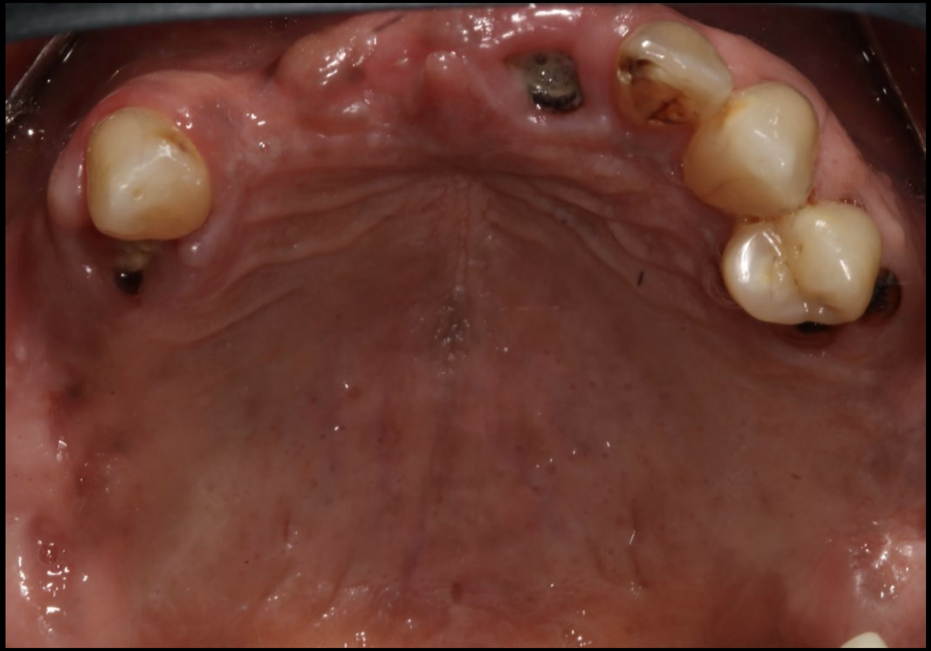

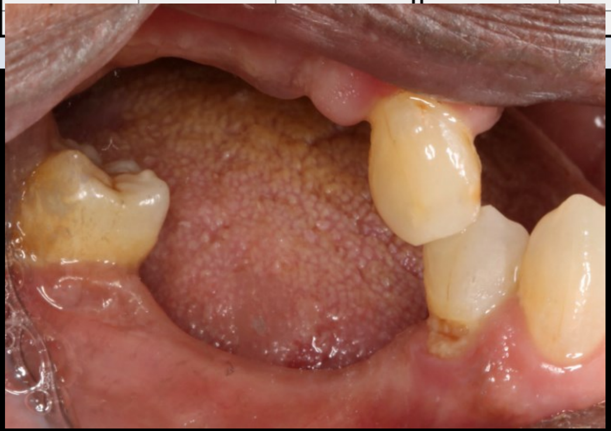

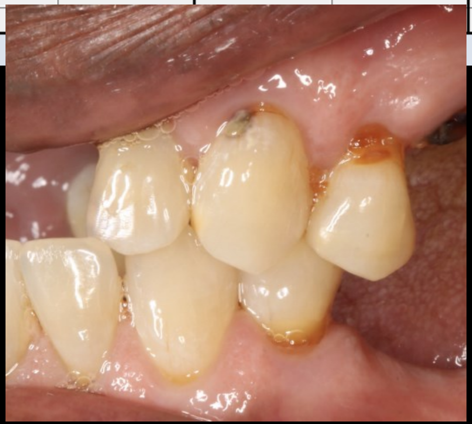

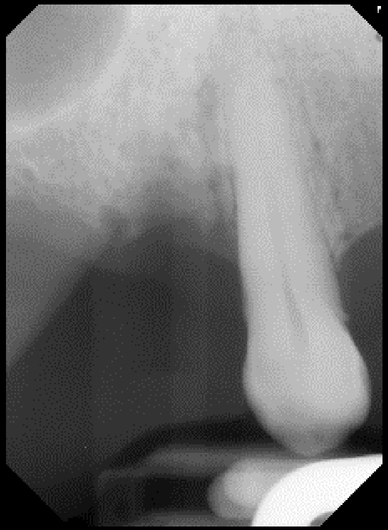

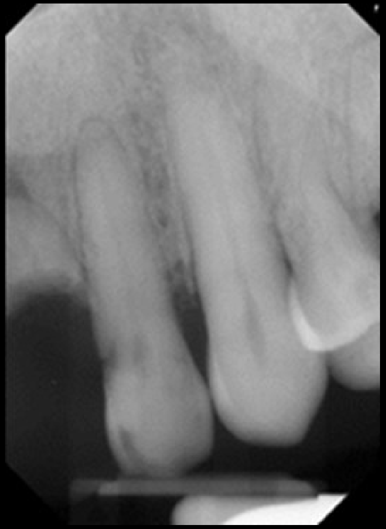

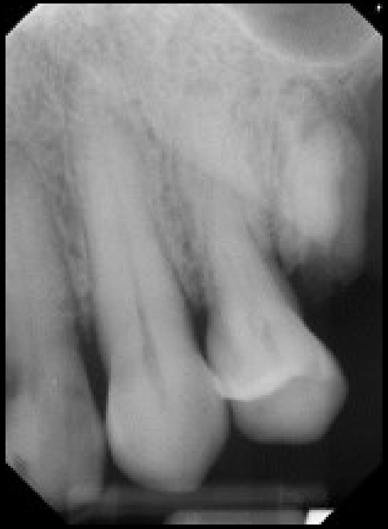

Lingual

		32			28			27			26			25			24			23			22			21	
Furc Inv		1																									
KER TIS																											
Bleed/S						B	B																		B		
CAL	3	5	2	3	3	5	4	3	3	3	2	2	2	2	2	2	2	2	4	4	4	3	2	3	4	3	3
PD	3	5	2	2	2	4	4	3	3	3	1	1	1	1	1	1	1	1	3	2	3	3	2	3	4	3	3
FGM-CEJ	0	0	0	1	1	1	0	0	0	0	1	1	1	1	1	1	1	1	1	2	1	0	0	0	0	0	0
PLQ/CALC	1	1	1	3	3	3	3	2	3	2	2	4	4	4	4	4	4	2	2	2	2	3	3	3	3	3	3

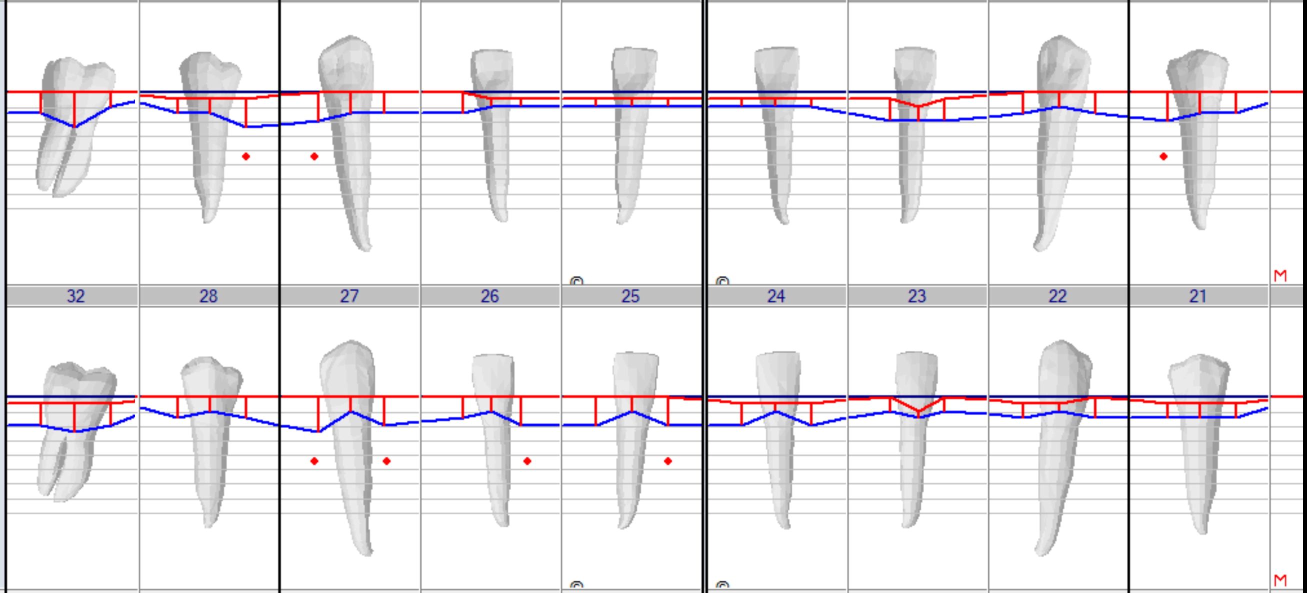

Facial

		32			28			27			26			25			24			23			22			21	
Mobil		1			2																						
Furc Inv		2																									
KER TIS		4			4			5			7			4			4			5			5			3	
Bleed/S							B		B			B			B												
CAL	4	5	4	3	2	3	5	2	4	3	2	4	4	2	4	4	2	4	2	3	2	3	2	3	3	3	3
PD	3	4	3	3	2	3	5	2	4	3	2	4	4	2	4	3	1	3	2	1	2	2	1	3	2	2	2
FGM-CEJ	1	1	1	0	0	0	0	0	0	0	0	0	0	0	0	1	1	1	0	2	0	1	1	0	1	1	1
PLQ/CALC	4	4	4	2	3	3	3	3	3	3	2	3	4	2	4	3	1	1	2	1	2	1	0	1	1	1	1

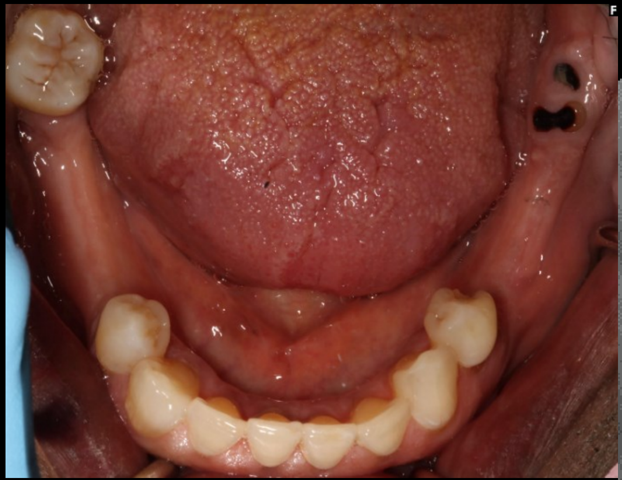

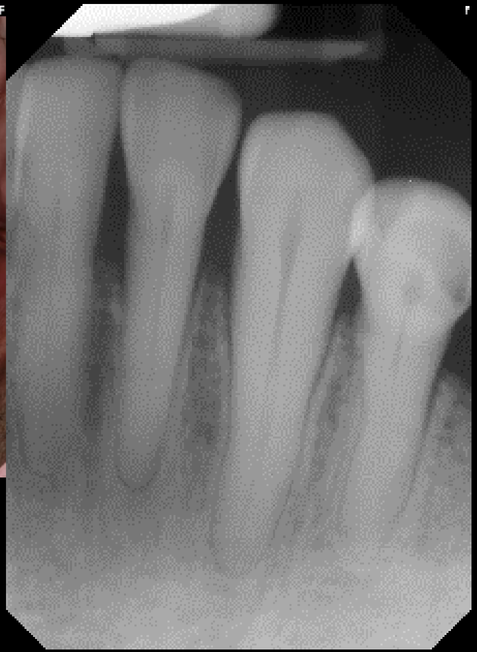

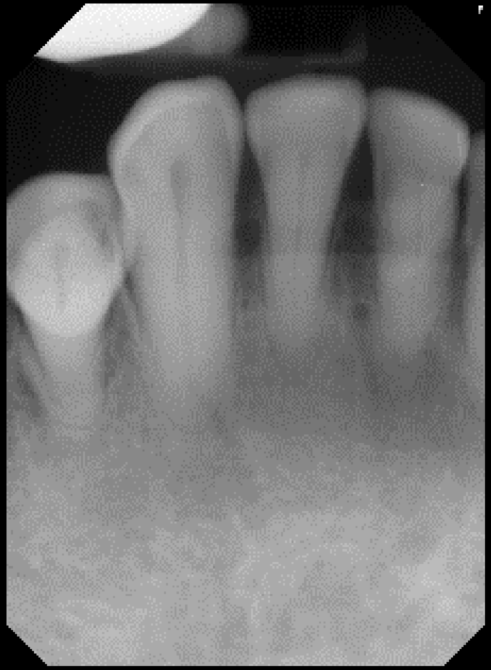

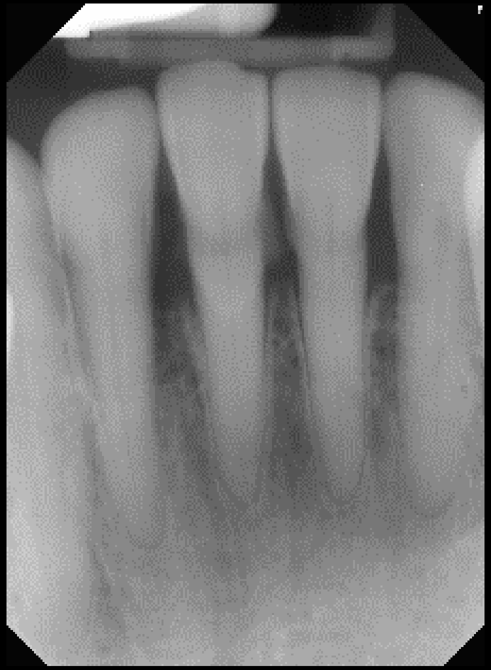

Case C

- A patient (age 50-59 years) visited the clinic.
- Chief Complaint: “ I want implant teeth “
- BP 115/73mmHg
- physician’s check up-3 years ago
- No-smoking
- ASA I
- #12 missing due to fracture 25 years ago
- #5 missing due to caries 5-6 years ago
- Regular dental check up every 6 months

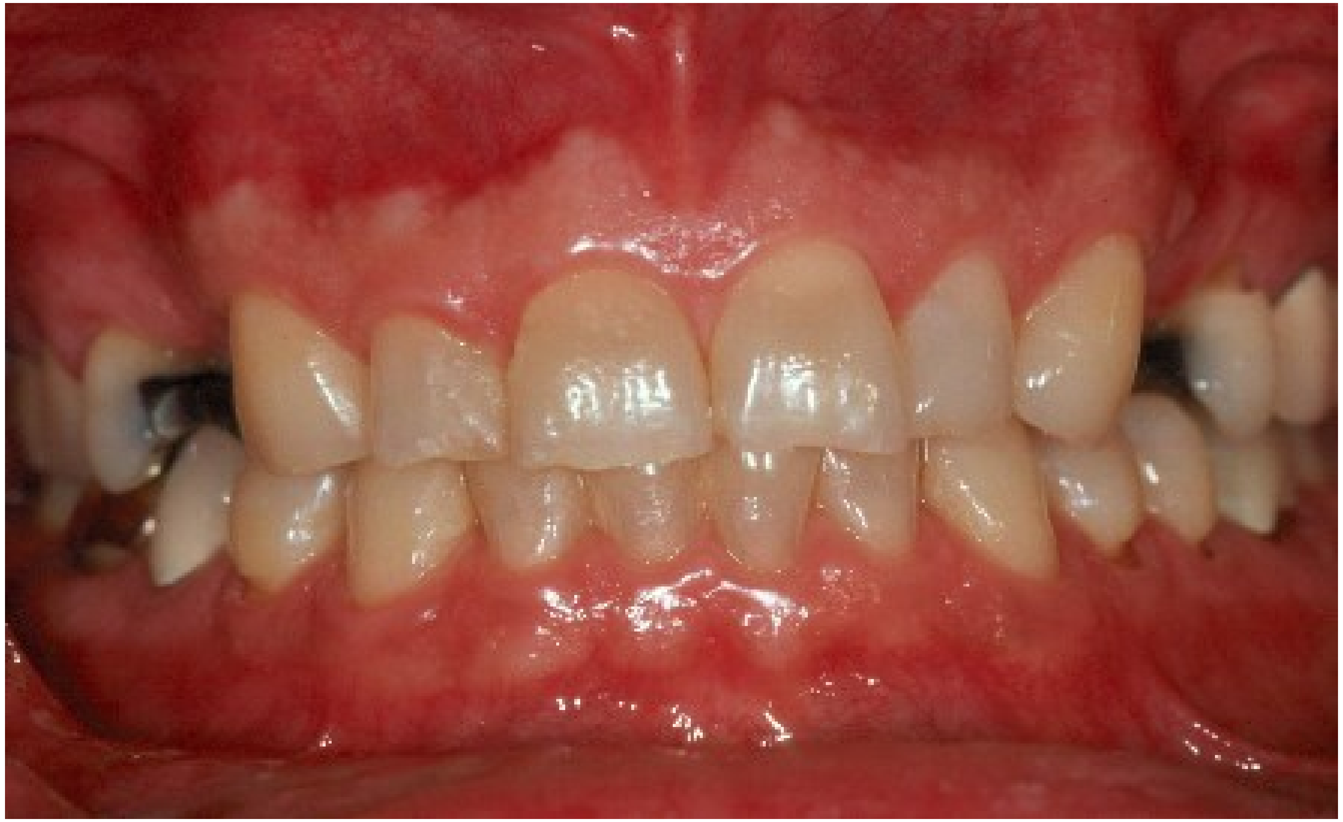

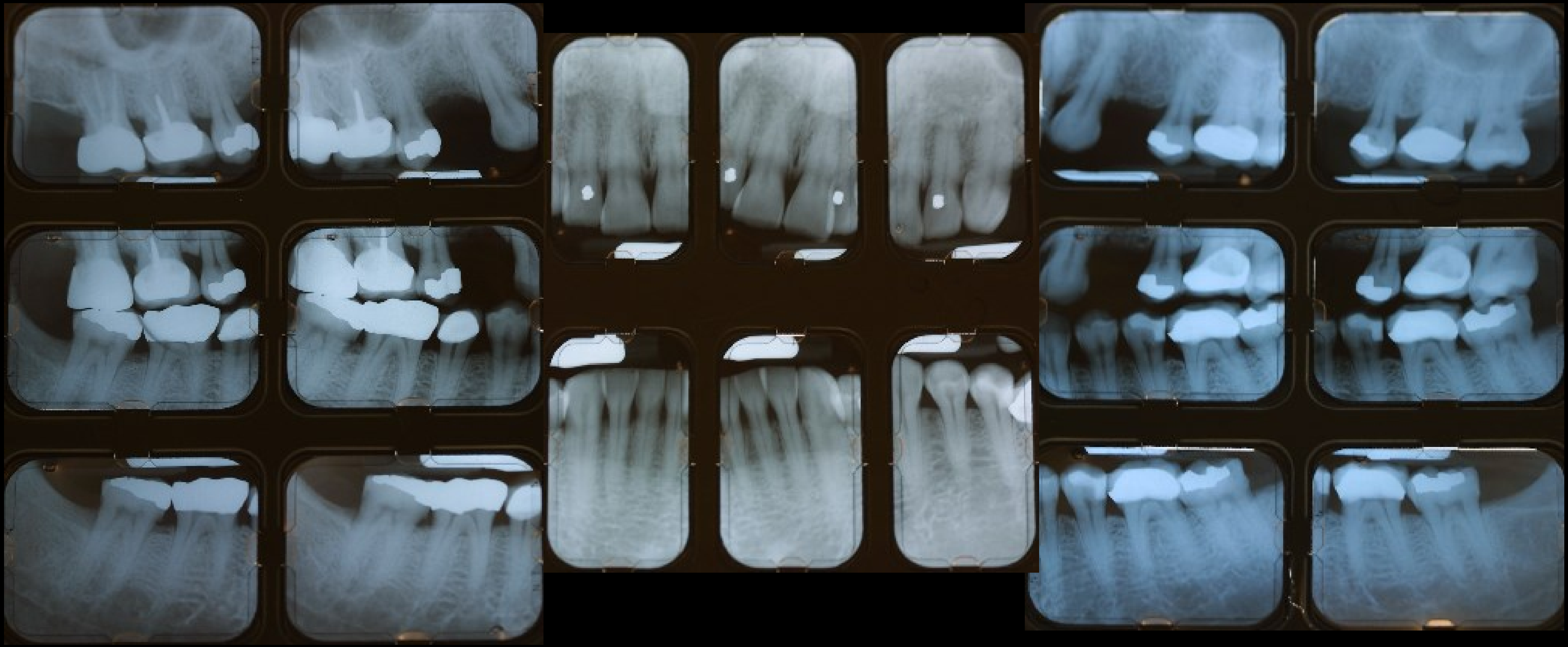

**Furcation**														
**FGM-CEJ (Facial)**	0 0 -1	0 1 0	01 0		-1 0 -1	-2 -2 -2	-1 -1 -1	0 0 0	0 0 0	0 0 0		0 1 0	0 1 0	0 00
**PD**	3 4 5	3 2 4	3 2 2		1 1 3	2 2 2	2 2 3	3 2 3	3 2 3	3 2 2		4 2 3	3 2 3	2 4 4
**CAL**	3 4 4	3 3 4	3 3 2		0 2 2	0 0 0	1 1 2	3 2 3	3 2 3	3 3 3		4 2 3	3 2 3	2 4 4
**Bleed/S**	B B B	B B B	B B B		B B	B B	B B B		B B B	B B		B B B	B B B	B B B
**Tooth #**	**2**	**3**	**4**	**5**	**6**	**7**	**8**	**9**	**10**	**11**	**12**	**13**	**14**	**15**
**Bleed/S**	B B B	B B B	B B B		B B B	B B	B B B	B B	B B B	B B B		B B B	B B B	B B B
**CAL**	4 3 3	3 3 4	4 3 3		3 3 3	2 2 3	3 2 3	3 2 3	3 2 3	3 2 3		3 2 3	4 3 3	3 3 3
**PD**	5 3 4	3 3 5	4 3 3		3 2 3	2 2 3	3 2 3	3 2 2	3 2 3	3 2 2		3 2 3	3 2 3	3 3 5
**FGM-CEJ (palatal)**	-1 0 -1	0 0-1	0 0 0		0 0 0	0 0 0	0 0 0	0 0 0	0 0 0	0 0 0		0 0 0	1 1 1	0 0 -2
**Mobility**	**0**	**0**	**1**		**0**	**0**	**0**		**0**	**0**		**1**	**0**	**0**
**Furcation**														

**Furcation**		**1**												
**FGM-CEJ (lingual)**	-1 0 -1	0 0 0	0 0 0	0 0 0	0 0 0	1 1 1	1 1 1	1 1 1	1 1 1	0 0 0	0 0 0	0 0 0	0 0 0	0 0 -1
**PD**	4 3 5	4 3 3	3 3 3	3 3 3	2 2 2	2 2 2	2 2 2	2 2 2	2 2 2	3 2 3	3 3 3	3 2 3	3 3 3	3 3 3
**CAL**	3 3 4	4 3 3	3 3 3	3 3 3	2 2 2	3 3 3	3 3 3	3 3 3	3 3 3	3 2 3	3 3 3	3 2 3	3 3 3	3 3 2
**Bleed/S**	B B B	B B	B B B	B B	B	B B		B B B	B B B	B B	B B	B B	B B B	B B B
**Tooth #**	**31**	**30**	**29**	**28**	**27**	**26**	**25**	**24**	**23**	**22**	**21**	**20**	**19**	**18**
**Bleed/S**	B B B		B B B		B B			B		B B	B B	B B	B B	B B
**CAL**	3 3 3	3 3 3	3 3 3	3 3 3	2 2 2	1 1 1	1 1 1	2 1 2	2 1 2	3 3 3	3 3 3	3 3 3	4 4 4	2 2 2
**PD**	4 3 3	3 2 3	3 2 3	3 2 3	2 2 2	2 2 2	2 2 2	3 2 3	3 2 3	3 3 3	3 1 3	3 1 3	3 3 3	3 2 3
**FGM-CEJ (facial)**	-1 0 0	0 1 0	0 1 0	0 1 0	0 0 0	-1 -1 -1	-1 -1 -1	-1 -1 -1	-1 -1 -1	0 0 0	0 2 0	0 2 0	1 1 1	-1 0 -1
**mobility**	**0**	**0**	**0**	**0**	**0**	**0**	**0**	**0**	**0**	**0**	**0**	**0**	**0**	**0**
**Furcation**	**1**	**1**											**1**	
